# “Grandmothers as gatekeepers”: The role of older women in addressing vaccine hesitancy in urban slum communities in Ibadan, Nigeria

**DOI:** 10.1371/journal.pgph.0005786

**Published:** 2026-01-06

**Authors:** Folusho Mubowale Balogun, Olubukola Christianah Omobowale

**Affiliations:** 1 Institute of Child Health, College of Medicine, University of Ibadan, Ibadan, Nigeria; 2 University College Hospital, Ibadan, Nigeria; 3 Department of Community Medicine, Faculty of Clinical Sciences, University of Ibadan, Ibadan, Nigeria; University of Oxford, UNITED KINGDOM OF GREAT BRITAIN AND NORTHERN IRELAND

## Abstract

Vaccine hesitancy is responsible for the resurgence of vaccine-preventable diseases in Nigeria due to suboptimal immunization as seen in urban slums. Older women are traditionally influential in infant health decision-making in African communities; however, their roles in addressing vaccine hesitancy remain underexplored. This study explored the perceived reasons for vaccine hesitancy and methods employed by older women to address vaccine hesitancy in selected urban slum communities in Ibadan, Nigeria. Narrative study design was adopted, and 22 focus group discussions were conducted with 175 older women from seven urban slum communities. Content analysis was used to analyze the data using the World Health Organization Strategic Advisory Group of Experts on Immunization (SAGE) ‘3 Cs’ model of vaccine hesitancy (complacency, convenience, confidence) and vaccine hesitancy determinant matrix. Perceived causes of vaccine hesitancy included poor knowledge about the importance of vaccines, parental nonchalance, low vaccination priority (Complacency), long clinic waiting times and vaccine stockouts (Convenience), mistrust of volunteers, poor healthcare workers’ attitude, immunization misconceptions, and previous negative experiences (Confidence). Older women addressed vaccine hesitancy through community mobilization, engagement with religious leaders, advocacy at the individual level, and by supporting trust in qualified health personnel. The older women demonstrated significant potential as key resources for addressing vaccine hesitancy. Leveraging their roles by formalizing their involvement in immunization programs could enhance the vaccination uptake in urban slum communities. Also, the existing approaches for handling vaccine hesitancy can be standardized to improve vaccination uptake.

## Introduction

Vaccine hesitancy is the delay in acceptance or refusal of vaccines despite the availability of vaccine services [1] and it is one of the greatest threats to global health [2]. It a complex phenomenon, which varies across cultures, with a wide range of manifestations (from delaying vaccines to selecting which vaccines to take, and complete vaccine refusal) [1]. Vaccine hesitancy undermines the coverage required for herd immunity. The herd immunity is the protection that a community has when the required minimum percentage of community members are vaccinated to prevent the transmission of vaccine-preventable diseases [3]. It is recognized as an individual behavior influenced by both internal (such as knowledge about vaccines) and external (e.g., sociocultural norms) factors [3]. There has been increasing interest in vaccine hesitancy because of the threats it poses to communities that have achieved herd immunity against vaccine-preventable diseases (VPD) using vaccines, and those with suboptimal vaccination [4]. This threat was evident during the introduction of the COVID-19 vaccine to combat the COVID-19 pandemic [5]. Also, there has been a resurgence of VPDs, such as diphtheria in Nigeria [6], and measles in the United States [7] because of vaccine hesitancy.

The complexities of vaccine hesitancy make it difficult to address it effectively. This is because the reasons for vaccine hesitancy are multifactorial, making effective strategies to be somewhat elusive [8,9]. Contextual variation and the many forms of hesitancy further complicate the problem [10]. In recognition of these complexities, the World Health Organization (WHO) Strategic Advisory Group of Experts on Immunization (SAGE) recommends the need to understand vaccine hesitancy and its determinants [1]. There are different strategies that have been deployed to address vaccine hesitancy, including attempts to improve knowledge about vaccines, reminders of appointments, and monetary incentives [11]. The most common strategy used to address vaccine hesitancy is to improve knowledge about vaccines, but this has shown inconsistent results [12]. There is a need to understand the underlying complexities that are involved in vaccine hesitancy to guide the design of context-specific strategies that can adequately address vaccine hesitancy. Also, for knowledge improvement-based interventions to succeed, there is a need to tailor the content of the message to reflect the realities of the community of interest and their unique information needs [12].

Despite the persistence of poor vaccine uptake in Nigeria, research on vaccine hesitancy is rare. The reasons for vaccine incompletion are mostly deduced from quantitative data and often interrogated using individual factors. The contextual reasons for vaccine refusal or incompletion are not considered, which gives rise to highly limited information on the reasons for vaccine hesitancy. The most common strategy for addressing vaccine hesitancy is to provide health information in the form of health talks about vaccines [13]. These talks are usually done by healthcare workers and mostly in health facilities and public spaces, but they are generally short, giving little or no room for questions from caregivers. The immunization uptake in urban slum communities has been inadequate despite access to immunization in urban settings [14], and this coverage is sometimes less than that in perceived disadvantaged rural communities. Children in urban parts of Nigeria are also more likely to experience missed opportunities for vaccination than those in rural areas [15]. However, the reasons for the vaccine hesitancy in urban slums are not clear. The importance of understanding the determinants of vaccine hesitancy in the urban slum communities in Nigeria cannot be overemphasized because it can lead to peculiar underpinning ideologies that can direct the design of interventions to effectively address the problem [16].

Older women are culturally recognized and highly revered influencers in the care of infants in many African communities [17]. They often play a major role in decision making for the uptake of healthcare services for infants, including immunization. Although they are not recognized in the formal health sector, their influence can be harnessed to promote infant immunization [18]. Urban slums are known for their poor health indices, which include suboptimal infant immunizations [14,19]. While rural areas readily receive support to improve their poor immunization coverage, poor immunization uptake in urban slums goes unnoticed and is not addressed as expected. SAGE encourages active community participation in the development of strategies against vaccine hesitancy to ensure that they address the context-specific determinants of vaccine hesitancy [8]. In addition, most successful interventions for vaccine hesitancy were tailored to address specific populations and vaccine concerns [13].

Therefore, this study was designed to explore older female caregivers’ perceived reasons for vaccine hesitancy and describe the available methods that they employ to address vaccine hesitancy in their communities. This study was part of a larger study that sought to improve infant vaccination timeliness and completion among infants from urban slum families through the support of older women caregivers [20].

## Methods

### Ethics statement

The study protocol was approved by the Oyo State Ethics Research Committee (AD 13/479/784), and University of Ibadan/University College Hospital Institutional Review Board (UI/EC/18/0206). Permission to conduct the study was sought from the executives of the Community Development Association of the study communities. Written informed consent was obtained from all older women who volunteered to participated in this study. Only numbers were used to identify responders during data collection and analysis. The women were assured of the confidentiality of the content of each discussion, and they were told that they could pull out of the study if they are no longer interested in participating, with no consequences.

### Study area

This study was conducted in seven purposively selected urban slum communities in Ibadan, the capital city of Oyo States in southwest Nigeria. They are inner-city slums in Atenda, Yemetu, Aladorin, Idi Ogungun and Oke Aremo communities in the Ibadan North local government area (which has the highest number of slum communities in Ibadan) [21]. The others were Oje and Beere from the Ibadan North East and South East local government areas, respectively. These communities are in the core traditional part of Ibadan City and are characteristically densely populated, with a high rate of poverty and inadequate basic amenities [22] and meet the criteria for the definition of slum according to the UN Habitat [23]. Healthcare services are delivered mainly through primary health centers, which also provide immunization services to the communities. They also accessed immunization services from a secondary health facility owned by the Oyo state government and a tertiary health facility owned by the federal government in the Ibadan North local government area.

### Study design and study population

A qualitative descriptive study with narrative study design was adopted and this was suitable in exploring the older women’s experiences with vaccine hesitancy, providing data about their perceived causes and management. The study population included older women (defined as women aged 35 years and above) who are already grandmothers and oversaw the care of infants in slum communities. This age was selected based on the early age that women from the least wealth quintile and little or no education (as seen in urban slums) in Nigeria have their first babies and subsequent grandchildren [24]. They mentor and support young mothers in caring for their infants. They are very influential in this regard, making their opinions about childcare respected. This culture is practiced in many African and other developing countries. Women must have lived in the communities for at least a year before the study to understand the dynamics of vaccine hesitancy in their communities before inclusion in the study.

### Data collection instrument

Focus group discussions were conducted to obtain data with a content and face validated interview guide (in the Yoruba language), which contained questions and probes based on earlier research and the researchers’ experiences with vaccine hesitancy. The interview guide was piloted among women in a community different from the study communities.

### Data collection procedures

Data collection commenced on 26 June 2018 and ended on 9 July 2018. Eligible participants were identified with the assistance of opinion leaders in the communities (which included the market leaders and the two ward chairmen). To mitigate gatekeeper bias, recruitment emphasized voluntary participation and confidentiality. Each participant was re-screened by the research team using the United Nations Human Settlement Program (UN HABITAT) criteria [23] to confirm that they live in urban slum setting before participation. Data were collected by four (three females and one male) trained research assistants with at least one degree (three of them had a master’s degree in public health). At the time of data collection, all the research assistants were full time research staff on the main project that determined how the involvement of older women could improve infant immunization coverage in urban slum communities of Ibadan. They all had prior experience conducting FGDs in community settings and completed refresher training on reflexive interviewing and safeguarding. All research assistants were unfamiliar to community members, except one female assistant who had previously lived in the area. This research assistant only took notes and timed the FGDs but did not facilitate any of them. All research assistants were able to speak the Yoruba language fluently. Data were collected in two halls within the communities. The study was introduced to each prospective participants and they were screened for eligibility. Thereafter, they were scheduled in groups for the focused group discussions (FGD) based on their availability. On the day of the interview, the study was re-introduced, and written informed consent was obtained from all participants. The FGDs were coordinated by a facilitator and were conducted in Yoruba. Each session was recorded using two digital recorders. There was a timekeeper who timed each FGD and a note taker who documented the sociodemographic characteristics of the participants as well as the discussions, including notes on group dynamics and non-verbal cues to contextualize transcripts during analysis. Member checking was conducted at the end of each FGD to ensure participants’ responses. Data collection continued until data saturation was attained (no new concept was seen in the content of the FGD when compared with the previous ones). This was achieved by comparing the content of each earlier interview with the next one. Each FGD lasted between 29–57 minutes. No refusals or dropouts were recorded during recruitment and data collection, and no repeat interviews were conducted. Only participants and the research team were present during FGDs; no non-participants attended.

### Data analysis

Content analysis guided by WHO SAGE frameworks (3Cs and determinants matrix) was used to analyze the data. Each recording was transcribed verbatim and translated from Yoruba into English. One of the researchers (FMB) selected three translated transcripts randomly and reviewed the translations (comparing them with the English version) to ensure accuracy. The scripts were critically reviewed by three coders and coded using inductive and deductive methods. The codes were then reconciled, with discrepancies resolved by discussions and consensus in a common meeting of the three coders. The perceived causes of vaccine hesitancy were categorized using ‘3 Cs’ model of vaccine hesitancy: complacency, convenience, and confidence [25]. Complacency refers to a low perception of susceptibility to the infection that the vaccine is intended to prevent, thereby downplaying the importance of getting the vaccine [26]. Complacency also includes low awareness or knowledge of vaccines [27]. When complacency is widespread in a community, the decision not to take a vaccine may be made passively rather than actively because of social norms [27]. Vaccination convenience refers to factors such as physical availability, affordability, willingness to pay, geographical accessibility, understanding of language (including health literacy), and appeal of immunization services that affect the uptake of immunization services [28]. Lastly, confidence refers to trust in the vaccine (effectiveness and safety), the health system (reliability of the services and the competence of health care workers), and the motivation of policymakers who make decisions about vaccines [28]. It also includes misinformation and views about vaccine-related risks [26].

The vaccine hesitancy determinant matrix of the World Health Organization (WHO) SAGE Working Group on Vaccine Hesitancy [28] was used as a framework to categorize the methods used by older women to address vaccine hesitancy. This vaccine hesitancy determinant matrix was designed based on the ‘3 Cs’ model of vaccine hesitancy, findings from earlier research, and the experiences of SAGE Working Group members. The matrix is presented in Table 1. Themes were derived through iterative comparison, integrating deductively anticipated categories with inductively emergent patterns. Major themes are presented with subheadings linked to the 3Cs framework. Minor themes and counter-narratives (e.g., concerns about baby pooling and distrust of volunteers) were also described. Samples of relevant quotes were given for each component of the ‘3Cs’ model and those of the determinants of vaccine hesitancy matrix.

**Table 1:** Working group on vaccine hesitancy determinants matrix [28].

Determinants	Content
**Contextual influences** Influences arising due to historic, socio-cultural, environmental, health system/institutional, economic or political factors	a. Communication and media environment
	b. Influential leaders, immunization program gatekeepers and anti- or pro-vaccination lobbies
	c. Historical influences
	d. Religion/culture/gender/socio-economic
	e. Politics/policies
	f. Geographic barriers
	g. Perception of the pharmaceutical industry
**Individual and group influences** Influences arising from personal perception of the vaccine or influences of the social/peer environment	a. Personal, family and/or community members’ experience with vaccination, including pain
	b. Beliefs, attitudes about health and prevention
	c. Knowledge/awareness
	d. Health system and providers – trust and personal experience
	e. Risk/benefit (perceived, heuristic)
	f. Immunization as a social norm vs. not needed/harmful
**Vaccine/vaccination – specific issues** Directly related to vaccine or vaccination	a. Risk/benefit (epidemiological and scientific evidence)
	b. Introduction of a new vaccine or new formulation or a new recommendation for an existing vaccine
	c. Mode of administration
	d. Design of vaccination program/Mode of delivery (e.g., routine program or mass vaccination campaign)
	e. Reliability and/or source of supply of vaccine and/or vaccination equipment
	f. Vaccination schedule
	g. Costs
	h. The strength of the recommendation and/or knowledge base and/or attitude of healthcare professionals

### Underlying data

The data set for this study can be found in Mendeley Data, https://data.mendeley.com/datasets/hyp5npbw9y/1 [29].

## Results

There were175 older women who participated in 22 FGDs, and their ages ranged from 35 to 80 years old. No variation was observed in the responses of the women based on their age. Table 2 summarizes older women’s perceived causes of vaccine hesitancy. In the complacency group, poor knowledge about vaccines and their importance were mentioned in every group for FGD. The nonchalant attitudes of parents and low priority alluded to vaccination were the other themes that were identified. For convenience, long waiting times was mentioned in all groups, and this was said to be more common in government-owned clinics. Vaccine stockouts emerged under convenience. Geographical access was not mentioned as a reason for vaccine hesitancy, as women in every group mentioned that immunization centers were readily accessible in their communities, as shown in the quote below.

**Table 2:** Perceived reasons for vaccine hesitancy by older women in Ibadan urban slum communities.

	Theme	Sample quote
Complacency	Poor knowledge of vaccines	It is people that know the importance of it that will immunize their children. FGD 4, p4
		…mostly it is the semi-illiterate and young people…that mainly oppose their children’s vaccination. FGD 17, p3
	Low priority for vaccination	some mothers use their business as a reason why they can’t take their children to the center for immunization FGD 20, p2
		for some people, they are lazy FGD 10, p2
		It is only nonchalant parents that will not take it…because they do not know its value. FGD 4, p8
Convenience	Long waiting time at the clinics	…the waiting time is quite long, you have to queue for a long time… you can even wait for 5 hours before being attended to… FGD 16, p5
	Vaccine stockouts	The vaccines given to a child at 9 months is not always available…I had to go the center multiple times for about 3 months before I got the vaccine for my child. FGD 7, p6
		My child now did not complete the vaccination schedule because it was not available at the immunization center; I went there repeatedly…when I was tired of going to the immunization center…I stopped going there to check. FGD 15, p6
	Pooling of babies for measles vaccine	…the vaccine at 9 months, mothers need to get to the facility at specific times because the nurses want many children to be at the center… so that when they open the vaccine it will not waste. I had to go to the center about 3 times before my child got the vaccine. FGD 6, p5
Confidence	Volunteers working with immunization officers.	I also told my children not to receive immunization from all the mobile immunization people…people that I use to talk to in the community are now part of the immunization team…these people know nothing about the process FGD 15, p7
	Previous bad experiences with immunization	…sometimes when a child is immunized, he would cry continuously for a long period of time and would be in discomfort…some parents don’t want their children to be in pain. FGD 17, p3
	Misconception	…people think that it causes sickness. Others think it causes delays in having more children. FGD 10, p6
		…there were a lot of myths then, about infant immunization. FGD 14, p3
	Poor attitude of health care workers	The nurses respond harshly to mothers and are not happy even when there are a lot of mothers and children in the center for immunization FGD 15, p4

Immunization centers are everywhere… The immunization team is now mobile. They are in the neighborhood, marketplaces, and everywhere… FGD 6, p4

The use of volunteers for National and State immunization days was a prominent theme in the confidence group. The other themes were the poor attitude of health workers at immunization clinics, misconceptions about immunization, and previous bad experiences with immunization. Religion was also mentioned in some groups as the reason why parents refused vaccination for their children, and the three common religions in Nigeria (Christianity, Islam, traditional religion) were mentioned as shown in the quotes below.

Many people say they are Christian/Muslim, and they do not want to immunize their children. FGD 18, p2

…like those Ìyá ifá (female ifá worshippers), there is no time they bring it that they agree to take it for the children… FGD 11, p4

Older women had different approaches for addressing vaccine hesitancy in different contexts, as shown in the following subsections.

### Addressing vaccine hesitancy from contextual influences

Different mechanisms were used to address vaccine hesitancy in slum communities. This includes community meetings where members are admonished to get vaccinated and report families who refuse vaccination. The community then send members to encourage families to be vaccinated. This system is described in the following quote:

Even in community meetings, we are often told to report mothers who do not immunize their children, and if any mother is reported, the community delegates will meet her, know the reasons for not immunizing her children... FGD 16, p2

In cases where such admonition is unsuccessful, the community pressurizes the family, including threats to get the parents arrested. This sometimes results in positive outcomes, as shown below.

Despite begging and imploring the man, he refused to allow his children to be vaccinated until he was threatened that he would be arrested…it was after this threat that he accepted for his children to be vaccinated. FGD 17, p4

Older women also facilitate the dissemination of immunization messages in various social and religious groups, as seen in the quote below.

In my own neighborhood, once they announce the day for routine immunization, I usually go to meet the Imam in the mosque to help us use their public speaker to announce to the people so that the people will be aware that we will come around for immunization... FGD 20, p6

Some of the older women were directly involved in the immunization process in their communities as volunteers during the national/state immunization days, members of Community Health Committees and Immunization town carriers. They often report families that refuse immunization following approved channels, as shown in the quote below.

Whenever we are in the community to provide immunization for children and…they refuse to allow their children to be vaccinated, we would report to our bosses, after which they will take the appropriate actions. FGD 17, p7

The older women also mentioned approaches used in the immunization clinics in their communities to address vaccine hesitancy, including health talks, educational wall posters, and songs sang at the clinics. These are represented by the following quotes:

…a child that is completely immunized will be strong and healthy just as the song we use to sing then “The child will be healthy, the child will be healthy, a fish does not get cold in the sea, the child will be healthy.” FGD 17, p7

At the hospitals, they normally paste pictures on the walls to warn the parents that if they do not immunize their children, they will look like a child with kwashiorkor. FGD 18, p2

### Addressing vaccine hesitancy from individual/group influences

Almost all older women had a positive perception of the importance of vaccination for infant wellness and survival. They mentioned protection from illnesses and a reduction in hospital visits as immunization benefits. These are presented in the following quotes:

Infant immunization is very good…in those days…immunization for infants was not common; polio, blindness, measles, were very common among young children, but with infant immunization, these kinds of diseases are no longer common. FGD 15, p8

Immunization is for protection…it decreases the frequency of hospital visits. FGD 20, p4

Older women also had witnessed the negative outcomes of not immunizing infants, which makes them admonish parents who refuse vaccination to allow their infants to be vaccinated, as shown in the following quotes.

I used to tell a woman to immunize her children…when three of the children had high temperatures…unfortunately only one of them survived…our people should go out and learn from the mistakes of others. FGD 10, p3

I could remember a man who said his mother had given birth to 10 children who had died before giving birth to the 11th child that survived. If there were immunizations, such things could not have occurred. FGD 18, p2

The older women also believed in the competence of the health care workers to deliver immunization services and that they were skilled in helping vaccine-hesitant parents, as shown below.

They speak gently to mothers and vaccinate children with great care FGD 15, p5

The mobile immunization team…even in some houses where they do not want the children to be immunized, they know what to do to immunize them FGD 16, p1

However, a few older women reported that some healthcare workers treat mothers harshly, which can lead to vaccine hesitancy, but they believed it was due to poor job satisfaction. This belief helped them to cope with these healthcare workers. This is illustrated in the following quote.

The nurses…used to nag…I know how they feel…there is no job satisfaction. FGD 15, p4

There were also instances in which older women assumed the responsibility of getting infants around them immunized. This is demonstrated by the older woman in the quote below when her daughter-in-law did not take the immunization of her baby seriously.

I shouldered the responsibility of taking the child for immunization, because she did not take it seriously…I go to their house early on the day of immunization. I will be the one that will wake them up. FGD 21, p6

Another woman also helped to source for a scarce vaccine for her grandchildren, as shown below.

…if my daughter/daughter-in-law calls me that the vaccine is not available at the center near her, I do go to the center close to me… to ask if the vaccine is available…I would tell her to come to the immunization center and get it for her child. FGD 014, p6

### Addressing vaccine hesitancy from Vaccine/vaccination specific issues

The presence of qualified health personnel increased older women’s trust in the immunization process, and this reduced hesitancy, as shown in the quote below.

It is very good if qualified personnel handle the immunization process for infants. FGD 15, p8

Health education regarding the vaccine to be obtained at each visit and its side effects also prevented vaccine hesitancy, as shown in this quote:

They also provide health education before the vaccine, tell us about the vaccine and its side effects… FGD 11, p8

In addition, health education through immunization campaigns by health workers also addressed some misconceptions about vaccines, as reported below by a woman who earlier alluded child’s death to vaccination.

This made me scared to immunize my children then; I did not immunize my first child…but after I heard a lot of information about the benefits of infant immunization, I began to immunize my children again... FGD 15, p6

## Discussion

The findings of this study highlight the complex interplay of factors contributing to vaccine hesitancy in Ibadan’s urban slum communities and the multifaceted roles older women adopt to address these challenges.

### Perceived causes of vaccine hesitancy

Our study identified several key determinants of vaccine hesitancy through the “3 Cs” model of vaccine hesitancy: complacency, convenience, and confidence. Under complacency, poor knowledge about vaccines and their importance emerged as a significant factor, this finding is consistent with previous research indicating that limited understanding of vaccine benefits contributes to hesitancy [30]. This supports the need for targeted educational interventions that address knowledge gaps within these communities.

Convenience barriers, particularly long waiting times and vaccine stockouts, are prominent concerns. These findings align with those of studies across other African settings, where health system challenges impede vaccination uptake [31]. Unlike research from rural settings where geographical access is often cited as a major barrier [32], our study found that physical access to immunization centers was generally favorable in these urban slum communities, representing a potential strength to build upon in intervention programs.

Confidence-related factors were significant determinants of hesitancy. The mistrust of volunteers observed in our study reflects findings from another Nigerian study in which the credibility of immunization personnel influenced parental decisions [33]. Clarity of the roles of volunteers during national/state immunization days can improve the trust of the older women in the vaccination process. Religious beliefs as a barrier to vaccination uptake also emerged as an important factor, consistent with research documenting the influence of religious doctrines on health-seeking behaviors across Nigerian communities [34]. However, only few groups mentioned religion as a reason for vaccine hesitancy and this may stem from the extrapolation of individual views of vaccine to rationalize vaccine rejection.

### Community-based approaches to address vaccine hesitancy

Our findings reveal several community-level mechanisms employed by older women to address vaccine hesitancy. The use of community meetings to encourage vaccination and the delegation of community members to persuade hesitant families demonstrates the importance of social influence in promoting health behaviors. This aligns with the theoretical understanding that social norms are powerful determinants of health decisions [27]. The involvement of religious leaders in disseminating immunization messages, as described by participants, further illustrates the integration of cultural and social structures in addressing hesitancy, similar to successful approaches documented in northern Nigeria [35]. The direct involvement of older women as volunteers, members of community health committees, and “immunization town criers” represents a valuable community communication resource that could be further leveraged in immunization programs. Their role in monitoring and reporting vaccine-hesitant families contributes to community surveillance systems that have been proven effective in other African settings [36]. The roles played by the religious leaders and older women showcased the existing culture towards vaccine hesitancy in the communities and community engagement can build on these existing social and cultural capital of the community to address vaccine hesitancy [37]. This also aligns broadly with current discourse in global health that calls for integration of locally relevant epistemic resources [38], such as the ones shown by grandmothers in our study. However, the use of threats or coercion are not appropriate strategies to address vaccine hesitancy. They do not only undermine the fundamental human right of autonomous decision making, but also result in stigma and social polarization, further widening health and social inequalities [39]. Threats and coercion can also fuel vaccine hesitancy by triggering anger and resentment when the freedom to choose is taken away [40].

### Individual influence and personal advocacy

The strong positive perception of vaccination among majority of these older women is the likely foundational element in their advocacy efforts. Their ability to reference personal experiences of vaccine-preventable disease consequences provides powerful testimonials that resonate with their communities and influence their decision to support immunization. This form of experiential knowledge sharing has been identified as an effective communication strategy in other African contexts [41]. Such experiences could also explain the proactive roles that the older women assume in facilitating vaccination, from physically taking children to immunization centers to navigating systemic challenges, such as vaccine shortages. These actions demonstrate their agency and commitment to child health, reflecting the important supportive roles older women traditionally play in childcare within African family structures [18], especially in patriarchal settings where older menopausal women are seen as synonymous with men [42]. The willingness of older women to compensate for the shortcomings of younger parents suggests a potential intergenerational intervention pathway that could be formalized in immunization programs.

### Health system approaches and trust building

The presence of qualified health personnel has emerged as a critical factor in building trust in immunization services. This finding aligns with literature emphasizing health worker quality in vaccine acceptance [43], while the value placed on pre-vaccination education underscores the role of transparent communication in reducing hesitancy [28]. While some participants reported negative interactions with healthcare workers, their understanding of potential underlying factors, such as poor job satisfaction, reveals a nuanced perspective that could facilitate community-health system collaboration. This understanding might serve as a bridge for improving relationships between communities and health facilities, addressing a common barrier identified in vaccination programs across sub-Saharan Africa [44].

### Implications for practice and policy

These findings have several important implications for improving the vaccination rates in urban slum communities. First, they suggested that older women can serve as effective agents for addressing vaccine hesitancy through their social influence, knowledge of local contexts, and commitment to child health. The formal incorporation of older women into immunization programs could enhance community outreach and monitoring systems. Second, the findings indicate the need for strengthening the health system to address convenience-related barriers, particularly waiting times and vaccine availability. Improving health workers’ attitudes through better working conditions and support might enhance service quality and build community confidence. Third, the successful community-based approaches identified, such as community meetings, religious leader involvement, and targeted education, provide models that can be standardized and scaled across similar settings. The multimodal nature of these approaches aligns with evidence suggesting that comprehensive interventions addressing the multiple determinants of hesitancy are most effective [45].

### Limitations and future research

This study has several limitations. The involvement of the community leaders in the identification of prospective participants could have introduced selection bias. Also, social desirability could have influenced the responses of the older women because they were interviewed in groups. The exclusive focus on older women provides valuable insights from their perspective but excludes the direct voices of younger parents who make vaccination decisions. Excluding younger caregivers may have introduced bias, as their perspectives on vaccine hesitancy could differ substantially from those of older women. This could create unintended consequences like conflict in decision making regarding infant immunization if policies are based solely on the older women’s perspectives. Future research should include multiple stakeholders to capture the full spectrum of hesitancy determinants and potential solutions. Additionally, while the qualitative approach revealed rich contextual information, quantitative studies are needed to measure the prevalence of the identified factors and evaluate the effectiveness of the reported approaches in improving vaccination rates.

Based on the results of the suggested research, the potential of formalizing the roles of older women in immunization programs through community health worker models, specifically tailored to leverage their influence and knowledge could be explored with experimental study designs. In addition, intervention studies testing community-health system partnerships that incorporate older women’s perspectives on vaccine hesitancy could generate evidence for scaling successful approaches. Lastly, the findings in this study may not be applicable to other urban slum settings even though older women involvement in childcare decision making is a common culture in Africa and many developing countries.

## Conclusion

This study provides valuable insights into the determinants of vaccine hesitancy in Ibadan’s urban slum communities and the roles played by older women in addressing these challenges. The findings underscore the vital role of older women in immunization programs, leveraging their social influence, commitment to child health, and contextual understanding of hesitancy. By integrating their perspectives and leveraging existing strategies within formal immunization programs, public health efforts may more effectively address the persistent challenge of vaccine hesitancy in disadvantaged urban communities. This approach aligns with the growing recognition of the importance of community engagement and culturally appropriate interventions to globally achieve immunization goals.

## Supporting information

S1 Checklist COREQ form. (DOCX)
